# Transcriptomic analysis of the anti-inflammatory effect of *Cordyceps militaris* extract on acute gouty arthritis

**DOI:** 10.3389/fphar.2022.1035101

**Published:** 2022-10-14

**Authors:** Chunwei Jiao, Huijia Liang, Li Liu, Shunxian Li, Jiaming Chen, Yizhen Xie

**Affiliations:** ^1^ Guangdong Yuewei Edible Fungi Technology Co, Ltd., Guangzhou, China; ^2^ Guangdong Yuewei Bioscience Co., Ltd., Zhaoqing, China

**Keywords:** gouty arthritis, inflammation, *Cordyceps militaris* extract, monosodium urate, transcriptomics

## Abstract

**Background:** Gouty arthritis (GA) is a common inflammatory disease that causes pain due to the deposition of monosodium urate (MSU) crystals into joints and surrounding tissues. Anti-inflammatory drugs have significant clinical anti-inflammatory and analgesic effects, but they have many side effects. *Cordyceps militaris* is an edible and medicinal fungus, and its extract (CME) has good anti-inflammatory and analgesic effects. This study aimed to investigate the anti-inflammatory effect of CME on GA and its underlying mechanism.

**Methods:** The effect of CME on the expression of related inflammatory factors and histopathological changes in the MSU-induced acute inflammatory gout model in rats was studied by ELISA and HE, and its anti-inflammatory mechanism was analyzed by transcriptome combined with RT-qPCR.

**Results:** CME significantly improved gait scores and joint swelling in GA rats, and reduced MSU-induced inflammatory cell infiltration. CME inhibited MSU-induced inflammatory responses by reducing the levels of pro-inflammatory factors TNF-α, IL-1β, IL-6, and Caspase-1 and increasing the anti-inflammatory factor IL-10. Transcriptome analysis showed that CME significantly altered inflammation-related cytokine pathways, and identified four major genes involved in regulation of inflammation, CCL7, CSF2RB, LIF, and IL-1β. In addition, RT-qPCR was performed to verify these differential genes.

**Conclusion:** CME significantly alleviated the inflammatory progression of GA and ameliorated the onset of GA. The underlying mechanism may be related to triggering the cytokine-cytokine receptor interaction signaling pathway to inhibit the activation of the inflammasome and regulate the immune system. And it regulates the inflammatory response induced by MSU crystals through the genes CCL7, CSF2RB, and IL-1β.

## 1 Introduction

Gout, a chronic disease of monosodium urate (MSU) crystal deposition, typically presents as acute inflammatory arthritis of the deposited crystals, progressing to more frequent attacks involving multiple joints and severe pain (Dalbeth et al., 2021). The most common symptoms include swelling, redness, pain, and joint fever (Rowczenio et al., 2017). Neglecting treatment can lead to an increased prevalence of other comorbidities, such as hypertension, diabetes, chronic kidney disease, and cardiovascular disease (Dalbeth et al., 2016). The initial treatment of gout aims to relieve the acute inflammation and pain of gout first, and then treat chronic gout to prevent recurring attacks. Therefore, acute gouty arthritis (GA), as the first clinical symptom, is the key to the treatment of gout. In addition, its main pathogenesis is that pathogenic crystals (MSUs) stimulate toll-like receptors (TLRs) and NOD-like receptor family pyrin domain-containing-3 (NLRP3) inflammasomes, which recruit monocytes, macrophages and cells such as synovial cells to release inflammatory factors through NALP3 inflammasome activity, leading to autoimmune disorders that promote inflammatory progression and tissue destruction (Dalbeth et al., 2021). The inflammasome is a key signaling platform for inflammatory responses, and active inflammasomes induce caspase-1 enzymatic activity, leading to inflammatory cell death and the maturation and secretion of inflammatory cytokines IL-1β and IL-18 (Spel and Martinon, 2020). IL-1β is a key inflammatory mediator that induces GA, stimulates NF-κB via TLR4 and TLR2, promotes inflammasome assembly, and regulates inflammatory cytokines (IL-1β, IL-6, IL-10, and TNF-α) (Martinon et al., 2006; Busso and So, 2010; Amaral et al., 2012; Andrés et al., 2015). To achieve anti-inflammatory and analgesic effects, the clinical treatment of acute GA currently relies primarily on nonsteroidal anti-inflammatory drugs, colchicine, and glucocorticoids (Dalbeth et al., 2016; Dalbeth et al., 2021). However, the long-term high-dose use of these drugs has limitations such as toxicity and side effects (Jayaprakash et al., 2007; Eleftheriou et al., 2008; Richette and Bardin, 2008). Among them, colchicine, the first-line treatment for gout, has toxic and side effects such as severe bone marrow suppression, renal failure, alopecia, disseminated intravascular coagulation, liver necrosis, diarrhea, seizures, and death (Roberts et al., 1987; Gottlieb et al., 2022). Therefore, there is an urgent need to develop new drugs that are safer and more effective in the treatment of GA.

Fungi have become a research hotspot in the field of edible medicine, with Phase I, II, and III clinical trials and widespread success in Asia for the treatment of various cancers and other diseases (Dubey et al., 2019; Panda and Luyten, 2022). *Cordyceps militaris* (L) Fr., belonging to Family *Cordycipitaceae,* Order *Hypocreales,* genus *Cordyceps,* class *Sordariomycetes,* Phylum *Ascomycota,* Kingdom *Fungi*, is an important edible and medicinal fungus which is parasitic on the cocoons and pupae of Lepidoptera larvae and is widely distributed all over the world (Sung et al., 2007; Singh et al., 2009). It has various biological activities such as anti-inflammatory, antioxidant, anti-aging, anti-tumor, and anti-proliferation (Liu et al., 1997; Nan et al., 2001; Zhan et al., 2006; Ji et al., 2009; Choi et al., 2014). And it is rich in various active chemicals such as cordycepin, adenosine, cordyceps polysaccharide, etc., which have the functions of enhancing immunity, lowering blood lipids, and anti-tumor (Wang and Zhang, 2021). Due to similar chemical characteristics and medicinal properties, *Cordyceps militaris* is increasingly used and considered as a substitute for Cordyceps sinensis (Chen et al., 2018; Zhang et al., 2019). *Cordyceps militaris* is sweet in taste, neutral in nature, beneficial to the lungs and kidneys, replenishes the essence, stops bleeding and resolves phlegm. It has been used for hundreds of years as a tonic in Chinese folk to treat impotence and assist in anti-cancer. Due to the great potential of natural products to develop new drugs, we chose *Cordyceps militaris* as a material for the treatment of acute gouty arthritis, based on its main component’s anti-inflammatory advantage through the NLRP3 inflammasome, and it has been used as a traditional herbal medicine to nourish the liver and kidney (Choi et al., 2014; Das et al., 2020; Yang et al., 2020). In addition, the liver and kidneys play important roles in uric acid reabsorption and metabolism. Gout is clinically characterized by hyperuricemia and urate crystal deposition, predisposing to the characteristic acute arthritis. At present, our team has carried out research on the treatment of hyperuricemia with *Cordyceps militaris* extract (CME) (Yong et al., 2016; Yong et al., 2018). Therefore, the main purpose of this study was to determine the effect and underlying mechanism of CME on MSU-induced acute gouty arthritis.

## 2 Materials and methods

### 2.1 Reagents and materials

*Cordyceps militaris* was provided by Hong Hao Biotechnology Co., Ltd., (Jiangmen, China). Cordycepin was purchased from Refensi Biotechnology Co., Ltd., (Chengdu, China). Colchicine was obtained from Sigma Chemical Co., (St. Louis, MO, United States). IL-1β, IL-6, IL-10, TNF-α, and CAS-1ELISA kits were purchased from Jiang Lai Biotechnology Co., Ltd., (Shanghai, China). Decalcification solution, hematoxylin and eosin were purchased from Sevier Biotechnology Co., Ltd., (Wuhan, China). TRIzol reagent was purchased from Thermo Fisher Scientific (Carlsbad, CA, United States). circRNAs and miRNAs, miRcute Plus miRNA First-Strand cDNA Kit and miRcute Plus miRNA qPCR Kit were provided by Tiangen Biochemical Technology Co., Ltd., (Beijing, China). The mRNA, GoScriptTM Reverse Transcription Mix Kit, and GoTaq^®^ qPCR Master Mix Kit were provided by American Promega Biotechnology Co., Ltd., Acetonitrile of HPLC grade was obtained from Merck (Darmstadt, Hesse, Germany). Pure water for HPLC was obtained from an ultrapure water system machine (PureLab, United States).

### 2.2 Preparation of CME

*Cordyceps militaris* was extracted twice with water at a solid-to-liquid ratio (1:10) at 100°C for 1 h–1.5 h. The vacuum concentration temperature is 45°C ± 5°C, the vacuum degree is −0.090 Mpa∼−0.095 Mpa, and it is concentrated to 12% ± 2% of Baileys. Dry at an inlet air temperature of 165°C–170°C and an outlet air temperature of 85°C–90°C. The resulting concentrate was purified on an macroporous resin (AB-8) column, first eluted with a 4% basic solution at a flow rate of 200 L/h, and then loaded onto an AB-8 resin column (φ20 × 180 cm × 2), the sample was loaded and washed with water at a flow rate of 2BV/h, then eluted with 10% ethanol at a flow rate of 2BV/h. Pump the alcohol cleaning solution to the single-effect concentrator, the concentration temperature is 45°C ± 5°C, the vacuum degree is −0.090 MPa∼−0.095 MPa, and the concentration degree is 15%–20% of the berry degree. The above-mentioned Cordyceps concentrate is spray-dried, the air inlet temperature is 165°C–170°C, the outlet air temperature is 85°C–90°C, the moisture content of the product is controlled to be ≤ 7%, and the concentration of soluble solids is 2%. The content of cordycepin in CME was 1% by high performance liquid chromatography.

### 2.3 Models and treatment

All experiments were conducted according to the “Guiding Principles for the Care and Use of Laboratory Animals”, and all procedures were reviewed and approved by Ethics Committee of Institute of Microbiology, Guangdong Academy of Sciences (approval number: GT-IACUC202106101).

Male Sprague-Dawley rats (5–6 weeks old, 200 ± 10 g) were purchased from Guangdong Medical Laboratory Animal Center (Certificate of Quality: SCXK-2018-0002). All rats were housed in a temperature-controlled room at 22°C ± 2°C, with 55% ± 10% relative humidity and a 12 h light–dark cycle. They were allowed food and water ad libitum for the duration of the study. After 1 week of adaptive feeding, rats were randomly divided into 4 groups, with 10 rats in each group: the gout group (Gout, 25 mg/ml MSU), CME-low group (CME-low, 0.135 g/kg/d), CME-high group (CME-high, 0.54 g/kg/d), colchicine group (Colchicine, 0.8 mg/kg) and control group (Control, distilled water). MSU was suspended in sterile phosphate-buffered saline and injected into the ankle joint after anesthesia in rats to model acute gouty arthritis. The establishment of this model is consistent with the method reported in previous study (Fan et al., 2021). In addition, this model has a high incidence of arthritis, peaking within 12 h, and spontaneous remission after 5–6 days, which completely simulates the pathogenesis of clinical acute gouty arthritis in humans (Coderre and Wall, 1987; Pineda et al., 2015).

The control and gout groups were given an equivalent volume of distilled water, and the CME group was given CME by intragastric administration for 9 consecutive days. After administration on day 10, all groups, except the control group, were given injection of 25 mg/ml MSU solution to the right ankle joint to induce acute gout model. Subsequently, the colchicine group was given 0.8 mg/kg colchicine-water solution. All animals were sacrificed at the end of the experiment. Serum was collected for enzyme-linked immunosorbent assay (ELISA). The ankle and foot joints were taken for histopathological analysis. The synovial fluid was removed from the ankle joints for transcriptomic analysis and quantitative real-time polymerase chain reaction (qRT-PCR).

### 2.4 Anti-inflammatory effect *in vivo*

#### 2.4.1 Joint swelling

The formation of ankle edema is one of the main symptoms in the evaluation of acute gout symptoms. We measured the ankle circumference at 0.5 mm below the ankle joint before and 2 h,4 h,6 h,8 h,10 h, and 24 h after MSU injection. Each measurement was repeated three times to obtain an average. The degree of ankle swelling (ASD) was calculated as follows: ASD (%) = [Ankle circumference after injection (mm)-ankle circumference before injection (mm)]/[Ankle circumference before injection (mm)] x 100%. Meanwhile, the ankle temperatures of these rats were measured with precision thermometers.

#### 2.4.2 Functional disorder evaluation

The evaluation standard of the dysfunction index refers to the method of scoring system (Coderre and Wall, 1987). Grade 0 dysfunction index evaluation: normal gait, all four feet are evenly on the ground; grade 1: left foot relaxed, toes open, mild lameness; grade 2: left hind foot bent, toes are touched on the ground, obviously lame; grade 3: left hind foot is completely off the land.

#### 2.4.3 ELISA

Blood samples were collected from the abdominal aorta 24 h after the MSU injection. The serum was separated for measurement of IL-1β, IL-6, IL-10, TNF-α, Caspase-1 using an ELISA kit according to its instructions.

#### 2.4.4 Histopathological analysis

The joint tissue was immersed in 4% paraformaldehyde and soaked in decalcification solution for 3 days. Then the tissue was fixed with a wax block and sliced, stained with hematoxylin and eosin (HE)solution after preparation, and the pathological changes of the joint were observed by light microscope.

### 2.5 Transcriptome of ankle joint

Total RNA was extracted from three randomly selected right joint tissue samples using TRIzol reagent. Libraries were identified and quantified by PCR amplification of enriched library fragments using an Agilent 2100 Bioanalyzer. Based on the Illumina HiSeq sequencing platform (Personalbio, Shanghai, China), the libraries were paired-end sequenced using Next-Generation Sequencing (NGS). After data filtering and quality assessment, the RNA-Seq data were compared with the reference genome with reference to the Ensembl database (http://www.ensembl.org/). The read count value for each gene was compared as the raw expression of the gene using HTSeq, and expression was normalized using TPM. mRNA expression level determined by FPKM > 1. Analysis of differential expression was performed using DESeq R (|log2 FoldChange| > 1, *p* value < 0.05). To gain insight into phenotypic changes, Gene Ontology (GO) and Kyoto Encyclopedia of Genes and Genomes (KEGG) functional and pathway enrichment analyses were performed by Metascape (http://metascape.org). GO covers three aspects, which describe the Molecular Function of genes, Cellular Component and Biological Process involved respectively. Go feature enrichment of differentially expressed genes (DEGs) and enriched pathways considered statistically significant were all defined by adjusted *p* < 0.05.

### 2.6 Quantitative real-time polymerase chain reaction analysis

Right joint tissue from the MSU rat model was used for total RNA extraction according to the manufacturer’s instructions for TRIzol. Total RNA was used to check quality and integrity shortly after reverse transcription to cDNA by real-time qPCR on the ABI 7500 PCR system (Applied Biosystems, CA, United States). For circRNAs and miRNAs, the miRcute Plus miRNA First-Strand cDNA Kit and the miRcute Plus miRNA qPCR Kit were used. PCR conditions were 95°C for 15 min, followed by 40 cycles of 94°C for 20 s and 60°C for 34 s. For mRNA, GoScript^™^ Reverse Transcription Mix Kit and GoTaq^®^ qPCR Master Mix Kit were used. PCR conditions were 95°C for 10 min, followed by 40 cycles of 95°C for 15 s and 60°C for 30 s.

### 2.7 Statistical analysis

All statistical analyses were performed using the GraphPad Prism software 8.0 program. Measurement data is presented as mean ± standard deviation, and the repeatability of each experiment is greater than or equal to 3. Normally distributed data were compared between multiple groups using one-way analysis of variance (ANOVA), and repeated measures and two-way ANOVA were used to analyze functional disorder evaluation, ASD and ankle temperatures, *p* < 0.05 was considered statistically significant.

## 3 Results

### 3.1 CME inhibits the onset of acute gout in rats

**FIGURE 1:**
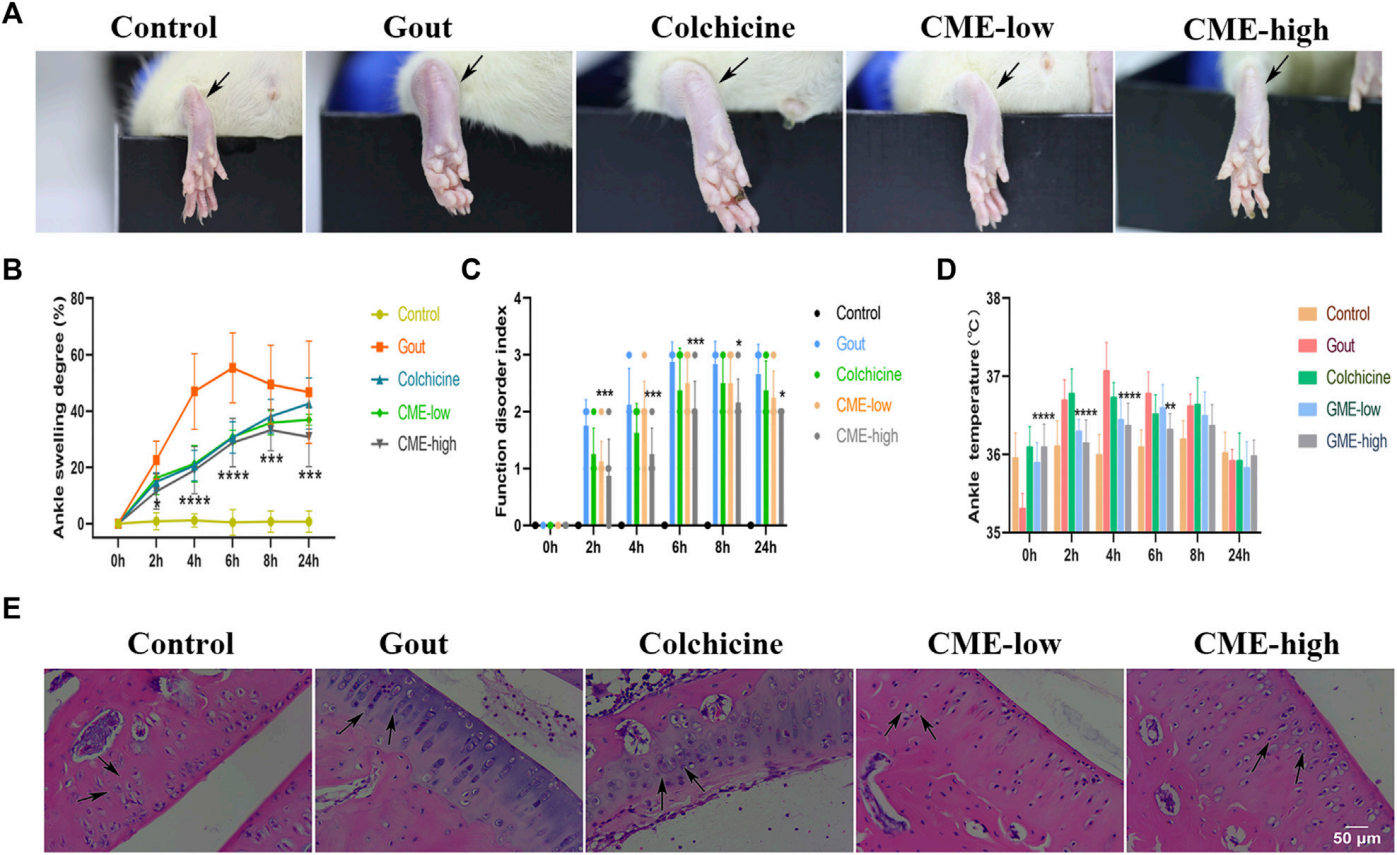
The effect of CME on MSU-induced naked joints in rats **(A)**Representative photographs of the ankle joints of rats after 24 h of MSU injection. **(B)**Ankle girth according to time after MSU crystal injection (*n* = 8/group). **(C)**MSU-induced ankle dysfunction index (*n* = 8/group). **(D)**The mean ankle temperature of MSU-induced gout model (*n* = 8/group). **(E)**Hematoxylin and eosin-stained sections of ankle joints after MSU induction for 24 h (200x). Data are expressed as mean ± SD (two‐way ANOVA), *p < 0.05, **p < 0.01, ***p < 0.001, ****p < 0.0001 vs. Gout.

Pain, stiffness, swelling, and inflammation in the joints and periarticular areas are the most common symptoms of MSU. To assess the recovery of these symptoms by CME, we found that the degree of heat, redness and swelling in the hind paws of the rats varied significantly between groups 24 h after MSU injection (Figure 1A). At the 6th hour, the gout group had the highest ASD, indicating that the swelling of the ankle joints was the most severe, showing a sharp increase from 0 to 6 h and a slow decline after 6 h–24 h (Figure 1B). However, at 0 h–6 h, compared with the gout group, the CME-high group significantly inhibited edema formation, restored foot function, and reduced redness and fever (*p* < 0.01), and compared with the colchicine group, the CME -high group had More effective prevention of gout flares in the short term (Figures 1B–D). These results suggest that CME ameliorates MSU crystal-induced acute gouty arthritis symptoms.

After 24 h, we selected rat ankles for sectioning and HE staining to study the tissue inflammatory state (Figure 1E). In the control group, the synovial tissue structure was clear, the cell morphology was normal, and there was no inflammatory cell infiltration. In the gout group, the density of interstitial cells increased, the infiltration of inflammatory cells was severe, and the synovial vacuolar hyperplasia was observed. However, these pathological changes were significantly reduced in both the CME-low and CME-high groups compared with the gout group. Especially after the treatment of CME-high group, the HE staining in the tissue synovium was more consistent with that of the control group. These results suggest that CME can effectively inhibit the inflammatory cell infiltration of MSU in naked rat joints and slow down the progression of synovial inflammation.

### 3.2 CME modulates inflammation in acute gout in rats

**FIGURE 2:**
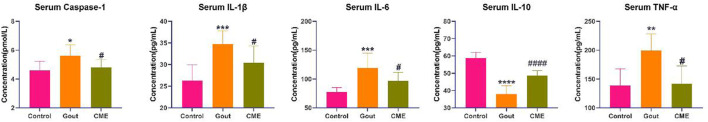
Analysis of the effect of CME on the levels of gout inflammatory factors by ELISA (*n* = 8/group). Data are expressed as mean ± SD (one‐way ANOVA), *p < 0.05, **p < 0.01, ***p < 0.001, ****p < 0.0001 vs. Control; #*p* < 0.05, ##*p* < 0.01, ###*p* < 0.001, ####*p* < 0.0001 vs. Gout.

By analyzing the effect of CME on GA in rats, it was found that the CME-high group had the best effect on GA. Therefore, the control group (Control), the gout group (Gout), and the CME-high group (CME) were used for subsequent specific mechanism analysis. MSU-induced acute GA triggers an inflammatory response by producing inflammatory mediators, which increases cytokine levels in tissues around the ankle joint. ELISA analysis (Figure 2) showed that the gout group significantly up-regulated the levels of IL-1β, Caspase-1, IL-6 and TNF-α, and significantly decreased the levels of IL-10, which were induced by MSU (*p* < 0.05). Compared with the gout group, the CME-high group significantly modulated the levels of these inflammatory factors (*p* < 0.05).

### 3.3 Effects of CME on the global transcriptomic profile of naked joints

**FIGURE 3:**
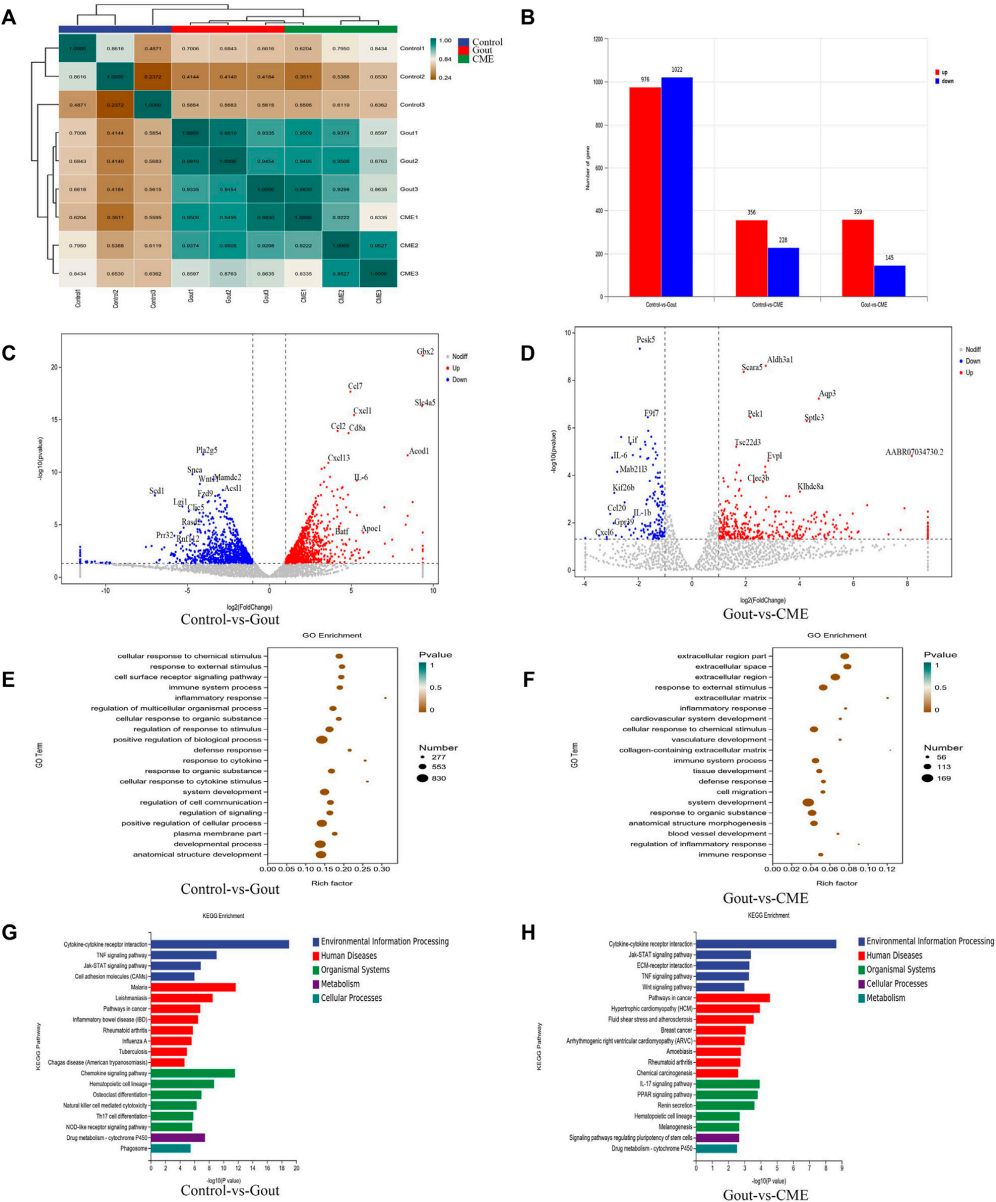
Effects of CME on the transcriptomic profile of MSU-induced acute gout. **(A)** Correlation heat map of differential genes between groups **(B)** Statistics of differential expression results **(C)** Volcano plot of differential genes between control and gout groups (*n* = 3/group) **(D)** Volcano plot of differential genes between CME and gout groups (*n* = 3/group) **(E)** Control and gout groups GO enrichment analysis **(F)** GO enrichment analysis of CME and gout group **(G)** KEGG enrichment analysis of control and gout group **(H)** KEGG enrichment analysis of CME and gout group.

With an expression level of FPKM > 1, 12,872 genes were successfully mapped and identified from RNA-Seq. The differential genes between the gout, control and CME groups are clustered together, with yellow colors representing significant correlations (between-group value < 0.8) and green representing low correlations. The heatmap showed that the differentially expressed genes were significantly separated between the CME group and the control group (Figure 3A).

We finally obtained a total of 1998 genes identified as DEGs in the control group VS gout group, of which 976 were up-regulated and 1,022 were down-regulated. A total of 504 genes were identified as DEGs in the CME group VS gout group, of which 359 were up-regulated and 145 were down-regulated. In the control group VS CME group, a total of 584 genes were identified as DEGs, of which 356 were up-regulated and 228 were down-regulated (Figure 3B). DESeq R analysis was performed on the condition that |log2 FoldChange| > 1, *p* value < 0.05, and the differential gene expression changes among the three groups were screened out. The genes CCL7, IL-6, GBX2, CXCL1, CD8A, CCL2, CXCL13, and SLC4A5, ACOD1 were significantly up-regulated in the control and gout groups. However, the genes PLA2G5, SNCA, WNT11, MADC2, SCD1, LGI1, CLIC5, RASD2, FZD9, and ACS11 was significantly downregulated in the control and gout groups (Figure 3C). After CME treatment, gout group genes AQP3, SPTLC3, PCK1, ALDH3A1, SCARA5, EVPL, TSC22D3, CLEC3B, KLHDC8A, and AABR07034730.2 were significantly up-regulated, gout group genes PCSK5, F9F7, LIF, IL-6, MAB21L3, KIF26B, CCL20, IL- 1β, CXCL6 and GPR39 were significantly down regulated (Figure 3D). The functions of the top 20 DEGs were analyzed based on the GO database and annotated with GO terms. The results showed that cellular response to chemical stimulus, response to external stimulus, cell surface receptor signaling pathway, immune system process, inflammatory response and regulation of multicellular organismal process were strongly expressed in biological processes; plasma membrane part, membrane microdomain and cell surface were dominant in cellular components; protein binding, cytokine binding and cytokine receptor activity were the main molecular functions (Figures 3E,F).

Through differential gene expression analysis of KEGG-enriched pathways, we concluded that in MSU-induced rats, the effect of CME on GA was significantly enriched mainly in the signaling pathway of cytokine-cytokine receptor interaction (*p* value < 0.05). Followed by chemokine signaling pathway, TNF signaling pathway, cancer pathway, IL-17 signaling pathway, rheumatoid arthritis, drug metabolism-cytochrome P450, inflammatory bowel disease (IBD) and cell adhesion molecule (CAM) enriched in signaling pathways (Figures 3G,H).

### 3.4 Confirmation of gene expression by RT-qPCR

**FIGURE 4:**
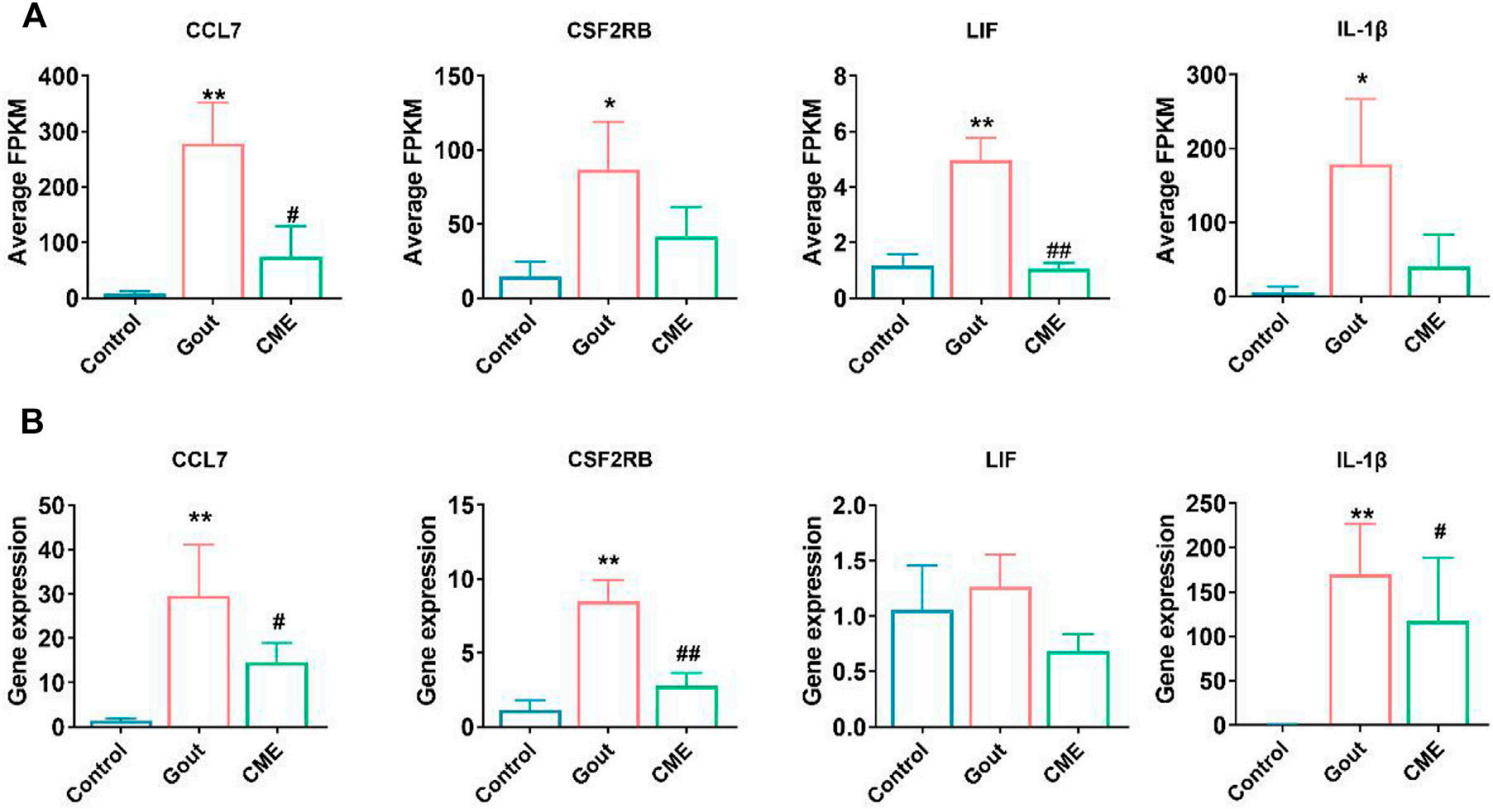
Comparison of relative expression levels between RNA-Seq and RT-qPCR results. **(A)** Detection of expression of four differential genes from RNA-Seq (*n* = 3/group). **(B)** Detection of expression of four differential genes by RT-qPCR (*n* = 3/group). Data are expressed as mean ± SD (one‐way ANOVA), *p < 0.05, **p < 0.01 vs. Control; #*p* < 0.05, ##*p* < 0.01 vs. Gout.

Focusing on the aforementioned GO terms and KEGG-enriched pathways, CME treatment of GA is closely related to the cytokine-cytokine receptor interaction signaling pathway. For further study, four down-regulated DEGs in the cytokine-cytokine receptor interaction pathway were randomly selected for RT-qPCR validation, including CCL7, CSF2RB, LIF, and IL-1β. The results of RT-qPCR showed that the expressions of CCL7, CSF2RB and IL-1β were significantly up-regulated in the gout group compared with the control group (*p* < 0.05). However, the expressions of 4 genes were significantly decreased after CME treatment compared with the gout group (*p* < 0.05) (Figures 4A,B). Therefore, the transcriptome sequencing data were consistent with the RT-qPCR gene results, indicating that the RT-qPCR results demonstrate the reliability of RNA-Seq data for gene expression profiling.

## 4 Discussion

Inflammation is one of the main causes of gout, because MSU crystals interact with cells, recruit a large number of infiltrating neutrophils, synovial cells, and tissue macrophages, resulting in immune system disorders, and the recruitment of immune cells leads to the production of inflammatory factors (Martin et al., 2009; Dinesh and RASOOL, 2017). In addition, clinical studies have shown that gout is prone to other complications, and the common link between these diseases is the presence of sterile inflammation (Pillinger et al., 2010; Ichikawa et al., 2011; Rahimi-Sakak et al., 2019). If inflammation is not treated promptly, a long-term immune program can have systemic consequences, leading to chronic inflammation and tissue damage (Cabău et al., 2020). Therefore, the anti-inflammatory effect plays a very important role in the treatment of gout. GA is an acute reaction triggered by MSU, which is one of the prominent clinical features of gout (Ragab et al., 2017). The GA attack is the pathogenic inflammatory response caused by MSU, during which IL-1β, IL-6, IL-10, TNF-α, Caspase-1 were all involved in the development of inducing and expanding inflammation (Das et al., 2020; Galozzi et al., 2021; Makoni and Nichols, 2021). NLRP3 and IL-1β have been identified as risk factors for gout, and it has been clinically confirmed that high levels of NLRP3, Caspase-1, IL-1β and IL-6 have been detected in serum and synovial fluid of patients (Son et al., 2013; Cavalcanti et al., 2016). Therefore, the expression of these proteins is an important factor affecting the onset of GA.

The biologically active compounds of *Cordyceps militaris* (total extracts, polysaccharides, and cordycepin) have significant immunomodulatory effects and can be used to treat tumors, allergies, viral infections, and as immunosuppressants for delayed-type hypersensitivity reactions (Lee et al., 2020). Among them, cordycepin has a protective effect on inflammatory damage in various diseases including acute lung injury, asthma, rheumatoid arthritis, Parkinson’s disease, hepatitis and atopic dermatitis (Tan et al., 2020). Cordycepin fights inflammation by regulating the release of IL-1β from the NLRP3 inflammasome (Yang et al., 2020). Studies have demonstrated that MSU crystals activate the NLRP3 inflammasome and release IL-1β to play a central role in the initiation of gout attacks (Dalbeth et al., 2021). Therefore, We mainly extract and isolate *Cordyceps militaris* extract, with cordycepin as the main active compound (Hu et al., 2015). Simultaneously, MSU-induced GA rat experiments were carried out to prove that CME (containing polysaccharide and cordycepin) effectively reduced the levels of IL-1β, IL-6, TNF-α and Caspase-1 pro-inflammatory factors and increased IL-10 anti-inflammatory factors in GA rats. Combined with the pathological changes of rat HE-stained joints, it was proved that CME significantly alleviated the inflammatory process. Furthermore, our results showed that CME can relieve joint swelling and fever in the short term, and the effect of restoring foot function was significantly better than that of colchicine. Overall, CME has great potential for the treatment of acute GA caused by MSU.

However, components of CME are complex, and the mechanism of treating GA is difficult to explain. Therefore, we used transcriptomics to elucidate the potential mechanism of action of CME in the treatment of GA. Our study concluded that the GO enrichment analysis of DEGs mainly involved immune system processes and inflammatory responses, and the KEGG analysis of DEGs identified several signaling pathways of inflammation-related cytokines, mainly including TNF signaling pathway, cancer pathway, IL-17 signaling pathway and rheumatoid arthritis. The present study demonstrated that these pathways were involved in the inflammasome-induced inflammatory response, which in turn leads to gout, indicating that the transcriptomic analysis of this study is consistent with previous findings (Latz and Duewell, 2018; Wree et al., 2018; Shin et al., 2019; Zhen and Zhang, 2019; Spel and Martinon, 2020). It was further found that the pathway after CME treatment in the KEGG analysis and the pathway of gout disease are involved in many of the same pathways to play their role, and the most influential one is the cytokine-cytokine-receptor interaction signaling pathway. To further dissect the mechanism of CME’s anti-inflammatory effect on GA, we analyzed DEGs belonging to the cytokine-cytokine-receptor interaction signaling pathway. These four DEGs (CCL7, CSF2RB, LIF, and IL-1β) were verified by RT-qPCR to promote the production and activation of inflammatory cytokines.

Inflammasomes aim for restoration of damage by initiating immune responses and repair mechanisms. When the activation of the inflammasome platform is unbalanced, its inflammatory effects can lead to arthritis (Spel and Martinon, 2020). We know that crystalline sodium urate is a damage-related molecule that can stimulate innate immune pathways and induce acute inflammatory responses, and the “inflammasome” is a core factor in activating inflammatory events (Martinon et al., 2002; Dinarello, 2019). Decreased levels of IL-1β inhibit msu-induced activation of NLRP3 inflammatory vesicles, impede maturation and secretion of inflammatory cytokines, thereby reducing the recruitment of neutrophils and other cells and regulating immune disorders (Liu-Bryan et al., 2005; Bauernfeind et al., 2009). LIF is a pleiotropic cytokine that modulates autoimmune responses by increasing T-cell numbers and neutrophil accumulation, and disrupts the balance of inflammasome platform activation by stimulating the release of cytokines from multiple cells, whose inflammatory effects lead to gout (Schainberg et al., 1988; Villiger et al., 1993; Janssens et al., 2015; Davis et al., 2018). LIF is induced in response to inflammatory stimuli of macrophages and switches macrophages from a pro-inflammatory phenotype to an anti-inflammatory phenotype. Further study LIF inhibits interferon gamma and granulocyte-macrophage colony stimulating factor (GM-CSF)-induced activation of STAT1 and STAT5 in macrophages, and inhibits macrophage motility through STAT3 and matrix metalloproteinase 9 (MMP9) (Hamelin-Morrissette et al., 2020). These pathways are mediated by ROS (Meier et al., 2017; Walter et al., 2020). MSU in turn activates NLRP3 inflammasome activity through ROS to produce IL-1β (Tschopp and Schroder, 2010). Our data showed that the transcript levels of CCL7, CSF2RB, LIF and IL-1β in MSU-induced GA rats were higher than those in normal rats, while the expression levels of these genes were significantly decreased after CME treatment. And RT-qPCR experiments verified the transcriptomic analysis. Therefore, we speculate that CME plays an important protective role in the regulation of inflammasome homeostasis during MSU-induced ventilation.

Inflammasomes are more commonly found in cells of myeloid origin, such as monocytes, macrophages, neutrophils, and dendritic cells. CCL7 is a chemokine that attracts monocytes and eosinophils, promotes the recruitment of many innate immune cell types, and thus mediates many inflammatory responses (Ben-Baruch et al., 1995). It is a chemokine that attracts monocytes and eosinophils and binds to CCR1, CCR2 and CCR3. Eosinophils are typical effector cells of allergic inflammation, and CCR3 mediates their migration to different chemokines to inflammation (Uguccioni et al., 1997). CCR2 potently regulates the recruitment of monocytes to sites of inflammation (Tsou et al., 2007). The CSF2RB is the common signaling subunit of the cytokine receptors for IL-3, IL-5, and GM-CSF (Croxford et al., 2015). It mediates IL-3, IL-5 and GM-CSF through cell surface receptors to stimulate eosinophil production, function and survival, thereby affecting inflammatory diseases (Begley et al., 1986; Lopez et al., 1986; Lopez et al., 1988; Rothenberg et al., 1988). Our results showed that CME treatment reduced the expression of inflammation-related genes and improved the inflammatory state of the joints of GA rats through the combination of cytokines and cytokines. In summary, CME treatment can effectively inhibit the inflammatory response of GA and modulate the interaction of inflammatory cytokines through CCL7, CSF2RB and IL-1β. Compared with colchicine and non-steroidal anti-inflammatory drugs (NSAIDs), which have clinical side effects, CME it has the potential for further development. In addition, our study demonstrated for the first time the anti-inflammatory and analgesic effects of *Cordyceps militaris* on acute gouty arthritis, providing pioneering evidence for gout treatment.

## 5 Conclusion

In conclusion, the effect of CME in relieving acute GA has been confirmed, and its molecular mechanism may mainly involve the cytokine-cytokine receptor interaction signaling pathway based on transcriptomic analysis and regulate inflammation through the levels of CCL7, CSF2RB and IL-1β reaction.

## Data Availability

The data that support the findings of this study are available from the corresponding author, CJ, upon reasonable request.
